# Congenital malformations among newborns admitted in the neonatal unit of a tertiary hospital in Enugu, South-East Nigeria - a retrospective study

**DOI:** 10.1186/1756-0500-5-177

**Published:** 2012-07-10

**Authors:** Herbert A Obu, Josephat M Chinawa, Nwachinemere D Uleanya, Gilbert N Adimora, Ikechukwu E Obi

**Affiliations:** 1 Department of Paediatrics, University of Nigeria/Teaching Hospital, P O Box 14587, Agbani Road PO, Enugu, 400001, Nigeria; 2 Department of Paediatrics, University of Nigeria/Teaching Hospital, Enugu, Nigeria; 3 Department of Community Medicine, University of Nigeria/Teaching Hospital, Enugu, Nigeria

**Keywords:** Congenital, Abnormalities, Newborns, Enugu

## Abstract

**Background:**

Congenital abnormalities are not uncommon among newborns and contribute to neonatal and infant morbidity and mortality. The prevalence and pattern of presentation vary from place to place. Many a time the exact etiology is unknown but genetic and environmental factors tend to be implicated.

**Methods:**

The objective of this study was to determine the prevalence of congenital malformations among newborns admitted in a tertiary hospital in Enugu, the nature of these abnormalities and the outcome/prognosis. For purposes of this study, congenital abnormalities are defined as obvious abnormality of structure or form which is present at birth or noticed within a few days after birth. A cross-sectional retrospective study in which a review of the records of all babies admitted in the Newborn Special Care Unit (NBSCU) of the University of Nigeria Teaching Hospital (UNTH), Ituku/Ozalla, Enugu over a four year period (January 2007-April 2011) was undertaken.

All babies admitted in the unit with the diagnosis of congenital abnormality were included in the study. Information extracted from the records included characteristics of the baby, maternal characteristics, nature/type of abnormalities and outcome.

Data obtained was analyzed using SPSS 13. Rates and proportions were calculated with 95% confidence interval. The proportions were compared using students *T*-test. Level of significance was set at P < 0.05

**Results:**

Seventeen (17) out of a total of six hundred and seven newborn babies admitted in the newborn unit of UNTH over the study period (Jan 2007-March 2011) were found to have congenital abnormalities of various types, giving a prevalence of 2.8%. Common abnormalities seen in these babies were mainly surgical birth defects and included cleft lip/cleft palate, neural tube defects (occurring either singly or in combination with other abnormalities), limb abnormalities (often in combination with neural tube defects of various types), omphalocoele, umbilical herniae, ano-rectal malformations and dysmorphism associated with multiple congenital abnormalities.

**Conclusions:**

The results of this study show that 2.8% of babies admitted to a Newborn Special Care Unit in a teaching hospital in Enugu had congenital abnormalities and that the commonest forms seen were mainly surgical birth defects and includes cleft lip/cleft palate and neural tube defects.

## Background

### Introduction

Congenital abnormality refers to any abnormality, whether genetic or not, which is present at birth [1]. It can also be defined as abnormality of physical structure or form seen at birth or few weeks after birth [2].

The aetiology of congenital abnormality may be genetic (30–40%) or environmental (5–10%) [3]. Among genetic causes, chromosomal abnormality makes up about 6%, single gene disorders about 25%, and multifactorial factors 20–30%. In about 50% of cases, the cause is not known [3].

Early intrauterine period (between the 3^rd^ and the 8^th^ week of gestation) is the vital period of life for the normal development of organs [4]. Any insult within that period may result in congenital abnormalities. It can further be argued that interventions within this period targeted at preventing insults (or removing the effects of insults) to the developing foetus will reduce the likelihood of an abnormality developing.

For instance, it is known that folate supplementation helps in the prevention of neural tube defects, especially in the first trimester. It is, however, observed that better maternal care and improved standards of living have little effect on the overall frequency of congenital malformations [5,6]. It is also noted that severe congenital anomalies diagnosed through age 5 years were observed to have a much higher incidence among children who weighed 2500 g or less at birth than among those who were heavier [7]. The reported incidence of congenital abnormality in newborns of non-consanguineous parents in a study by Naderi is 1.66% as compared to 4.02% among babies of consanguineous parents [8]. Consanguineous marriages have been described as an important factor contributing to increase in congenital malformations [8]. This is influenced by the degree of relatedness between the spouses i.e. first cousins, double first cousins, second cousins etc.

Consanguinity, however, is not a common practice among the Igbos who are the indigenous and predominant inhabitants of Enugu and environs but is occasionally noted among migrant people of Hausa-Fulani extraction, especially among the Fulani nomads seen in the area.

The prevalence of congenital abnormalities ranges from 1% to over 4% depending on the place and population studied [9,10]. For instance; it ranges from 1.07% in Japan (where the general population was considered) to 4.3% in Taiwan [5] (in a hospital based study).

Congenital anomalies involving the brain are reported to have the highest incidence at 10/1000 live births compared to heart at 8/1000, kidneys at 4/1000, limb at 1/1000 while all others have a combined incidence of 6/1000 live births [11]. Reported incidence is higher in black children than in whites [7]. It increases approximately three and a half fold for blacks and five folds for whites between 6 days and five years of age [7].

Congenital abnormality plays a major role in morbidity and mortality of children [12]. However, the treatment and rehabilitation of these children with congenital abnormality is very costly, hence the need to identify causative and risk factors and prevent them early [12], where possible. The birth of an infant with major malformations whether diagnosed ante-natally or not evokes an emotional parental response [12]. Early recognition of anomalies is important for planning and care. Parents are likely to feel anxious and guilt on learning of the existence of a congenital anomaly and require sensitive counseling [13].

In the tropics, malnutrition and infections are main causes of infant morbidity and mortality while in the temperate zones, cancer, accidents and congenital abnormalities are the key causes of infant morbidity and mortality [12].

Prevalent studies of congenital anomalies are useful to establish baseline rates, to document changes over time and to identify clues to aetiology. They are also important for health services planning and evaluating antenatal screening in populations with high risk. The study is also important as it may help to raise the awareness of surgical paediatric intervention and to emphasize the loss of babies with congenital abnormalities.

We are not aware of any study of this nature from Enugu or South-East Nigeria in general. In addition, the University of Nigeria Teaching Hospital (UNTH) moved to its permanent site at Ituku-Ozalla four years ago and since then no work has been done on the prevalence and pattern of presentation of congenital abnormalities in newborns in the area. This study was thus designed to bridge this gap with a view to determining the prevalence of congenital abnormalities among newborns admitted in the Newborn Special Care Unit of the University of Nigeria Teaching Hospital, (UNTH), Ituku-Ozalla, Enugu and the different types of abnormalities that are prevalent It is hoped that this will add to the body of knowledge available on these disorders and may stimulate further research in the area on the subject.

## Methods

The aims and objectives of this study were to determine the prevalence of congenital abnormalities among babies admitted at the Newborn Special Care Unit of the UNTH, Ituku-Ozalla, Enugu; to describe the different forms of abnormalities seen among these babies; to determine the various birth characteristics, maternal characteristics and outcome of congenital abnormality in UNTH, Ituku -Ozalla. For purposes of this study, congenital abnormalities are defined as obvious abnormality of structure or form which is present at birth or noticed within a few days after birth.

The study was conducted at the Newborn Special Care Baby Unit (NBSCU) of the University of Nigeria Teaching Hospital (UNTH), Ituku/Ozalla, Enugu. The unit was established in 1975 to offer special care to at risk and ill newborn babies. The hospital was then located at her temporary site within the city (Enugu) centre. In January 2007, the hospital was re-located to its permanent site at Ituku/Ozalla, about 15 km away from Enugu metropolis.

The NBSCU provides care for babies born within and outside the hospital and also receives referrals from different parts of Enugu, the rest of Enugu State and surrounding states. Enugu State of Nigeria has a population of about 3 million people according to the national census of 2007; the surrounding states of Abia, Anambra, Benue, Ebonyi, Delta, Imo and Kogi have populations ranging from 2 to five million people.

The unit is currently staffed by 4 consultants, 4 senior registrars, 4 registrars, 3 house-officers and eighteen nurses. Facilities for incubator care, intubation, assisted ventilation, supplemental oxygen administration, phototherapy and exchange blood transfusion are available in the unit in addition to other basic newborn services. Facilities for genetic testing are not available in our centre and are thus not offered to babies treated in the unit.

A cross-sectional retrospective study in which a review of the records of all newborns admitted in the Newborn Special Care Unit (NBSCU) of the UNTH, Ituku-Ozalla over a four year period (January 2007 and April 2011) was undertaken. The folders (case files) of these babies were retrieved from the hospital records department and examined individually by the investigators. Data collection was done with structured forms designed for the study. The diagnosis of congenital abnormality was based on clinical evaluation and ultrasound examination (as documented by doctors in the patients’ folders). Patient’s history, including antenatal history, history of exposure to teratogens and family history of consanguinity were obtained from these folders. Further information obtained include maternal age, type of delivery, gestational age and type of congenital abnormality. The prevalence rate was estimated as a per cent of the total number of babies admitted in the unit within the period of the study (Number of babies with congenital abnormalities/total number of babies admitted in the hospital for the duration of study). Data was analyzed using SPSS 13. Rates and proportions were calculated with 95% confidence intervals. The proportions were compared using students *T*-test. Level of significance was set at *P* < 0.05. Ethical approval the for this study, and consent to publish the clinical data obtained in the study, have been sought for from the Ethics and Research Committee of the University of Nigeria Teaching Hospital, Ituku-Ozalla, Enugu, Nigeria.

## Results

A total of six hundred and seven babies were admitted in the Newborn Special Care Unit (NBSCU) of the hospital over the study period. Seventeen of these were found to have congenital abnormalities of various types, giving a prevalence of 2.8% (of total admissions in the neonatal unit over the study period).

Table 1 shows the characteristics of babies with congenital abnormalities: the mean birth weight (in kg) is 3.06 ± 0.48SD with a minimum value of 2.4 kg and maximum of 4.25 kg; mean occipitofrontal (Head) circumference(in cm) is 33.11 ± 3.12 SD, minimum of 27 cm, maximum of 37 cm and Mean length (in cm) is 48.6 ± 3.0 SD, minimum value of 41 cm, maximum of 52 cm.

**Table 1:** Distribution of Birth characteristics

	*N [%]*
*Gender*	*N = 17*
Male	8 [47.1]
Female	9 [52.9]
*Birth Weight [Kg]*	*N = 12*
<2.5	5 [29.4]
2.6– 4	6 [35.3]
>4	1 [5.9]
*OFC [cm]	*N = 17*
26 – 30	3 [17.7]
31 – 35	10 [58.8]
36 – 40	4 [23.5]
*Length [cm]*	*N = 12*
<50	7 [41.2]
>50	5 [29.4]

*OFC occipitofrontal (head) circumference.

Table 2 shows maternal characteristics with a mean maternal age (in years) of 29 ± 5 SD, minimum of 20 years, maximum of 37 years.

**Table 2:** Maternal Birth Characteristics

*Characteristic*	*N [%]*
*Maternal Age [yrs]*	*N = 15*
20 – 25	3 [17.7]
26 – 30	5 [29.4]
31 – 35	6 [35.3]
36 – 40	1 [5.9]
*χ*2 = 3.933, df = 3, P = 0.269	
*Gestational age at birth [weeks]*	*N = 17*
37	1 [5.9]
39	3 [17.6]
40	13 [76.5]
*Mode of delivery*	*N = 17*
Spontaneous Vertex	14 [82.4]
Caesarean Section	3 [17.6]
*ANC attendance*	17 [100.0]
*Trimester of ANC commencement*	*N = 14*
First	4 [23.5]
Second	8 [47.1]
Third	2 [11.8]
*Drugs ingested during pregnancy*	
*[Multiple choice allowed]*	
Herbs	3 [17.6]
Routine drugs	14 [82.4]
Other OTC drugs	2 [11.8]
*Family History of Birth deformity*	
No	13 [76.5]
No response	4 [23.5]

Table 3 shows the types of congenital abnormality seen in these babies with outcome.

**Table 3:** Description of the congenital abnormalities and their outcome

Congenital abnormality observed	N = 17	Outcome
	n [%]	
Ano-rectal Malformation [?Imperforate Anus]	1 [5.9]	
		Treated
Left Cleft-lip/Palate	2 [11.8]	NR
Multiple Congenital Anomaly [?Patau Syndrome]	1 [5.9]	
		DAMA
Myelomeningocele/Hydrocephalus/Bilateral	1 [5.9]	
Talipes Equinovarus		Treated
Myelomeningocele/Hydrocephalus/Umbilic	1 [5.9]	
al Hernia/Hypertrophied clitoris		DAMA
Myelomeningocele/Obstructed	1 [5.9]	
Hydrocephalus		Treated
Myelomeningocele [?Ruptured]/	1 [5.9]	
Hydrocephalus		Treated
Myelomeningocele-Ruptured/	1 [5.9]	
?Hydrocephalus		Treated
Myelomeningocele-Ruptured/	1 [5.9]	
Hydrocephalus		DAMA
Occipital Cranial Bifidum/Ruptured	1 [5.9]	
Encephalocele		NR
Occipital Encephalocele –Holoproencephaly	1 [5.9]	
[?Lobar/Semi-lobar Encephalocele]		NR
Occipital Encephalocele	1 [5.9]	T/C
Occipital Encephalocele/Hydrocephalus	1 [5.9]	NR
Occipital Encephalocele [Ruptured]	1 [5.9]	NR
Omphalocele	1 [5.9]	Treated
Omphalocele Minor	1 [5.9]	T/C

*DAMA* discharged against medical advice.

*T/C* treated with complications.

## Discussion

The prevalence of congenital abnormalities of 2.8% obtained in this study is similar to the findings of Asindi et al. [4]and Naderi et al. [9] In a hospital-based study in Saudi Arabia, Sallout et al. [14] obtained a prevalence of 2.79% which is also comparable with our finding. The observed similarities in prevalences with studies that looked at populations not similar to ours is difficult to explain. However the fact that both studies were done in referral institutions where major congenital defects are admitted may offer some explanation for the observed similarities. Sawardekar [15] however noted a prevalence of 1.2% in a regional hospital in Oman. The Oman study cited above concentrated on minor abnormalities alone and this may have accounted for the lower prevalence rate obtained in the study.

The prevalence rate of 2.8% obtained in this study does not reflect the picture in the general population as this was purely a hospital based study with no attempt whatsoever to obtain a sample that would be representative of the general population. Be that as it may, it is possible that a community based study or one taking into account all deliveries occurring in the larger society may yield a higher prevalence. In our part of the world, for instance, some babies with congenital abnormalities brought to teaching or specialist hospitals do not present to the neonatology unit but are seen at other specialist units such as paediatric surgery unit or neuro-surgery unit etc. and a study conducted at the neonatology unit per se as is the case in this work may not be able to “capture” these other babies. Some that are born outside the hospital with congenital abnormalities are not taken to hospitals for care but are taken to traditional healers or other alternative practitioners while some are just left at home to their fate. Some that are brought to peripheral hospitals may not be referred to tertiary or teaching hospitals for care. Needless to mention that majority of these babies are not well managed and a good number die or are left with avoidable complications.

The mean maternal age (in years) of those with congenital abnormality is 29 ± 5. Grag and colleague [16] also noted a high occurrence of congenital abnormality among women who are between 33 and 39 years of age. Tennat and co-workers noted that high pregnancy rates among mothers in this age range could account for this [17].

All the seventeen mothers whose babies had congenital abnormality attended antenatal clinic. However most of the mothers that attended ante-natal clinic enrolled during the second trimester. As mentioned earlier, early intrauterine period (between the 3^rd^ and the 8^th^ week of gestation) is the vital period of life for normal development of organs [6] and insults or deprivations occurring during this period may predispose to congenital malformations. It is plausible, therefore, to argue that lack of ante-natal care or delay in commencing ante natal-care (with attendant inability to receive, or delay in receiving, some necessary micronutrient and other supplementation such as folic acid), especially in the early period of pregnancy when organogenesis begins, may have contributed to the occurrence of congenital abnormalities described in this study. It is noteworthy that only defects requiring intervention were noted in this study, this is because less severe defects unrelated to the infant’s illness and admittance were perhaps not recorded.

Brain abnormalities (meningocoele, myelocoele and encaphalocoele) are observed to occur more frequently than other abnormalities in this study. This is in keeping with the findings of Kumar et al. [10]. The reasons adduced above, especially lack of folic acid supplementation, may explain the increased occurrence of these disorders in our series. However the reason for this predominance could also be less related to folic acid deficiency than to the fact that these congenital abnormalities are severe enough to require quick intervention but usually survive for surgery. Another possible explanation for this is that these disorders may be perceived by the parents as being severe, with an increased likelihood of their seeking medical support compared to other abnormalities such as musculoskeletal abnormalities which may be perceived as being less severe. This may explain the contrary observation in the study by Christianson et al. [18] who noted higher musculoskeletal abnormalities than neural tube defect among black population.

It may be pertinent at this stage to highlight the fact that antenatal screening for congenital abnormalities is not routinely offered in our hospital; this practice needs to be put in place as early screening may detect such abnormalities and may influence decision to seek medical help earlier.

All the mothers whose babies had congenital abnormalities booked for antenatal care in the second trimester or later. Ignorance and poverty may have accounted for this. Ambe et al. [19] in north-east Nigeria noted that about 90% of women who had children with birth defects did not attend ante-natal clinic but preferred to deliver their babies outside the teaching hospital with traditional birth attendants in attendance.

### Limitations

A retrospective, cross-sectional study of this nature is bound to be faced with a number of challenges, and expectedly so, as the investigators are not fully “in-charge” of the processes. Firstly, retrieving patients’ folders from the hospital records department (which is yet to be fully computerized) was a rather herculean task; some of the folders retrieved contained inadequate information and this affected the quality of the study. In addition, a hospital based study of this nature, especially one restricted to only a section of the hospital as is the case in this instance, cannot be said to reflect truly what obtains in the general population. A prospective, community based study is thus desirable.

## Conclusions

The results of this study show that congenital abnormalities occur in 2.8% of newborns admitted in the neonatal unit of the University Of Nigeria Teaching Hospital, Ituku-Ozalla, Enugu and that the commonest forms seen are cleft lip/cleft palate and neural tube defects. The prevalence rate obtained in this study, however, may not reflect the true situation in the general population for reasons adduced in the discussion above but gives a clue to the existence of the problem and could serve as a stimulus for further studies on the subject.

## Misc

Herbert A Obu, Josephat M Chinawa, Nwachinemere D Uleanya, Gilbert N Adimora and Ikechukwu E Obi are contributed equally to this work.

## Competing interest

The authors hereby declare that we have no competing interests.

## Authors’ contribution

All the authors made substantial intellectual contributions to this study. HAO was involved in the conception, design, data collection, interpretation of results, preparation of the manuscript, revision of the article at various stages and preparation of the final draft. JMC, NDU and GNA made substantial contributions in the design, data collection, interpretation of the results, preparation of the manuscript, revision and preparation of the final draft. IEO undertook analysis of the data and also participated in the interpretation of the results and preparation of the final draft.
